# Study on the fusion of sports and medicine in China from 2012 to 2021: A bibliometric analysis *via* CiteSpace

**DOI:** 10.3389/fpubh.2022.939557

**Published:** 2022-09-16

**Authors:** Si-Jing Tu, Chen Jin, Bu-Tong Chen, An-Ying Xu, Chao Luo, Xiao-He Wang

**Affiliations:** ^1^School of Public Health, Hangzhou Normal University, Hangzhou, China; ^2^School of Public Health and Management, Guangxi University of Chinese Medicine, Nanning, China; ^3^School of Humanities and Social Sciences, Guangxi Medical University, Nanning, China; ^4^School of Artificial Intelligence and Information Technology, Nanjing University of Chinese Medicine, Nanjing, China; ^5^Shanghai Key Laboratory of Psychotic Disorders, Shanghai Clinical Research Center for Mental Health, Shanghai Institute of Traditional Chinese Medicine for Mental Health, Shanghai Mental Health Center, Shanghai Jiao Tong University School of Medicine, Shanghai, China

**Keywords:** CiteSpace, bibliometric analysis, data visualization, fusion of sports and medicine, health policy

## Abstract

**Objective:**

To analyze the research hot spots and frontiers of studies on of the fusion of sports and medicine (FSM) in China in recent decade *via* CiteSpace.

**Methods:**

Relevant publications related FSM published from January 1, 2012 to December 31, 2021 were obtained from the China National Knowledge Infrastructure (CNKI) database. CiteSpace software was used to analyze the amount of publications, institutions and keywords using standard bibliometric indicators.

**Results:**

A total of 729 publications on FSM were identified, and 688 qualified records were included in the final analysis. Between 2012 to 2021, the number of publications showed a trend of growth, albeit with certain fluctuations. The authors of these publications were mainly from universities or colleges with sports background. The institution leading the study was the Beijing Sport University (*n* = 20), the most prolific (*n* =12) and most-cited (224 times) author was Guo JJ from Capital University of Physical Education Sports. The journal with most publications on FSM was *Contemporary Sports Technology* (*n* =74). The analysis of keywords showed that the “FSM” had the highest frequency (*n* = 269), “integration of sports and medicine” had the strongest citation bursts (4.82), “national fitness” had the highest centrality (0.97) in recent decade, and 15 clusters of keywords were produced by log-likelihood ratio (all silhouette value >0.9).

**Conclusion:**

The findings of this bibliometric study analyse the current status and trends in the FSM in China, which may help to identify hot topics, explore new study directions for scholars and policymakers in the future.

## Introduction

According to the *China Country Assessment Report on Aging and Health* released by WHO in 2015, the prevalence of chronic diseases in China will increase by at least 40% by 2030 (1). The Chinese government is fully aware of the great threat of chronic diseases confronting national health, which may induced by insufficient exercise and unhealthy lifestyle, and it is quite necessary to introduce effective policies to promote health management in China. Although sports had been used in the prevention, treatment and rehabilitation from disease for thousands of years, the concept of “integration of sports and medicine” (ISM) was first proposed in America in the late nineteenth century and put into practice until the late twentieth century. The American College of Sports Medicine proposed “Exercise is Medicine” (EIM) for the first time in 2007, and now EIM has developed from an academic idea into a practical model, which has been widely implemented and developed in more than 100 countries around the world (2).

The term “ISM” first appeared in Chinese publications in 2007, it was used to refer to the combination of community exercise and city health services. On the contrary, “integration of medicine and sports” referred to take medical treatment as the core and sports as the auxiliary. In 2012, Chinese Sport Science Society and Chinese Center for Disease Control and Prevention joined the EIM project, marking the in-depth integration of public health and sports science research, and strongly promoting the formulation and implementation of the policy of ISM in China. From then on, more and more studies under the guidance of ISM or EIM theory were conducted, however, with different expressions, such as “exercise and health,” “physical education and medical education,” and “sports and medicine combination.” In 2016, the “*Healthy China 2030*” planning outline issued by the Chinese government proposed that “to strengthen ISM and non-medical health intervention, promote disease management and implement strategic task of establishing health service model of ISM,” which indicated that the concept “ISM” had the first unified expression and elevated to the political heights in China (3). In 2021, a new concept of the “fusion of sports and medicine (FSM)” was proposed in China, which is inherited the concept of EIM and it is more comprehensive than ISM (4). The FSM aims to change the traditional method of health intervention with treatment priority, promote the idea and new method of health intervention with prevention priority, and achieve “preventive treatment of disease.” However, the definition of “FSM” is still in the exploratory stage. Many scholars in China believe that FSM, in broad sense, refers to the choice of fitness means and ways by the combination of sports and medicine; in narrow sense, it means a method to combine physical exercise habits and medical care knowledge with the goal of establishing “Healthy China” and improving national fitness of Chinese people (5).

To carry out a new model of health management with Chinese characteristic, the scholars has conducted many studies on FSM with key words such as chronic diseases, health services and community. However, the research hot spots and frontiers of studies on of the FSM in China in recent decade is unknown, a thorough review is needed. Bibliometrics is one of the most important quantitative index evaluation methods, mainly based on the quantity and quality of academia publications, and make objective evaluations *via* the characteristics of content and structure. With the development of computer technology, large professional databases and various software, e.g., CiteSpace, help to realize the automation, intelligence and visualization of bibliometrics, and greatly improve the evaluation efficiency. CiteSpace software is run in a Java environment and was first created by Chen MeiChao, a professor working in the College of Information Science and Technology at Drexel University in the United States (6). Through the main procedural steps of CiteSpace, including time slicing, thresholding, modeling, pruning, merging, and mapping, the visualization knowledge map can be yielded. Central concepts of CiteSpace includes burst detection, betweenness centrality, and heterogeneous networks, which can help to timely visualize the research status, hot spots, and frontiers. In this study, we use bibliometric analysis to identify hot spots and frontiers of FSM in China in recent 10 years, then explore new study directions of academic study so as to contribute to policy making in the future.

## Methods

### Data sources and search strategies

The publications were obtained online through the China National Knowledge Infrastructure (CNKI) database. CNKI database, the largest and the most comprehensive online academic database in China, containing various academic resource such as journals, doctoral and masters' dissertations, conference proceedings, newspapers, yearbooks, statistical yearbooks, ebooks, patents, and standards. The reference resource was limited to “Journal” in order to sort out other type of resources. Because the official expression of “ISM” and “FSM” was proposed in China in 2016 and 2021, respectively, the keywords in this field were inconsistent in Chinese, let alone in English. Therefore, the publications were searched by keyword “sports and medicine” (体 医), and removed the unrelated ones manually. Due to the poor quality and little quantity of publications before 2012, the time span was selected from January 1, 2012 to December 31, 2021. All data were acquired on April 1, 2022, so as to avoid the prejudice caused by the database update. The duplicated, unrelated, conference abstracts or corrigendum documents were removed.

### Bibliometrics and visualization analysis

Full records of these publications were downloaded from the CNKI database, saved in Refwork format, and then imported into Citespace V5.8 R3 (64-bit) for further analysis. Nodes in different maps represent authors, institutions or keywords. Size of nodes indicates the frequency of occurrence, and color of nodes indicated the occurrence years. Besides, nodes with purple trims suggests high betweenness centrality, which are often identified as hot spots or turning points in a field. The line connecting the nodes represents the keywords are related in the publications, the thickness the line, the closer the relationship between keywords.

The parameters were set as follows: (1) time slicing from 2012 to 2021 at 1 year per slice; (2) the selection uses a modified g-index in each slice: k = 25, which means that data were extracted on the top 25 results for each time slice; (3) the node type was set as author/institution/keyword; (4) choosing “Pathfinder” and “Pruning the merged network” for Pruning parameters area to simplify the network and highlight its important structural features. The remaining parameters were the default settings. The top 10 keywords with the strongest citation bursts were identified and presented using Microsoft Excel 2019.

## Results

### Bibliometric analysis of the temporal distribution

**Figure 1:**
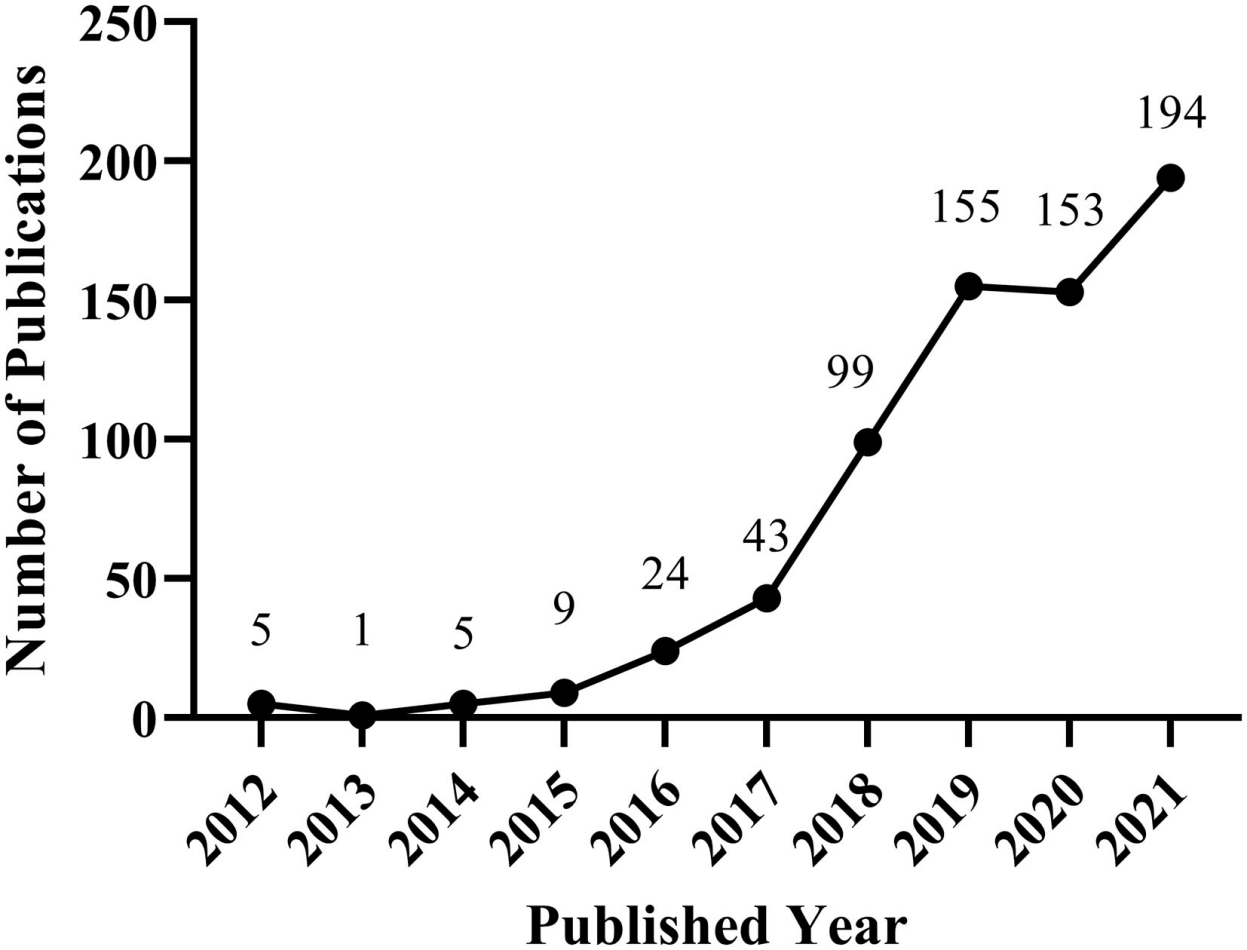
The number of publications on fusion of sports and medicine in China from 2012 to 2021.

Between 2012 and 2021, 727 publications on FSM were identified in CNKI database, and 688 qualified records were included in the final analysis after removing the invalid ones (*n* = 39). As shown in Figure 1, the number of publications on the topic of FSM in China showed an generally growing trend in last decade. The first stage was the exploration stage. From 2012 (*n* = 5) to 2015 (*n* = 9), the concept of “ISM” began to attract scholars' attention, but with few publications. The second stage is the development stage. From 2016 (*n* = 24) to 2021 (*n* = 194), the concept of “FSM” showed up and the amount of publications had an explosive increase but fluctuated slightly in 2020.

### Bibliometric analysis of the of disciplines and institutions distribution

To identify the disciplines leading the research on FSM in China, the analysis of discipline distribution was conducted. The 688 publications can be categorized in more than 40 disciplines, and some of which belong to two or more disciplines. The top 5 disciplines were Sports (*n* = 540), Study of Health Policies and Laws (*n* = 224), Medical Education and Interdisciplinary Medicine (*n* = 44), Special Medicine (*n* = 28), and Higher Education (*n* = 26) (Table 1). It indicated that the research on FSM is led by sports and assisted by medicine in China.

**Table 1:** Discipline distributions of publications on the fusion of sports and medicine in China between 2012 and 2021 [*n* (%)].

**Rank**	**Disciplines**	**Publications**
1	Sports	359 (50.78)
2	Study of medical and health policies and laws and regulations	217 (30.69)
3	Medical education and medical fringe disciplines	38 (5.37)
4	Special medicine	21 (2.97)
5	Higher education	21 (2.97)
6	Chinese politics and international politics	12 (1.70)
7	Secondary education	11 (1.56)
8	Traditional Chinese medicine	10 (1.42)
9	Endocrine gland and systemic diseases	9 (1.27)
10	Clinical medicine	9 (1.27)

**Figure 2:**
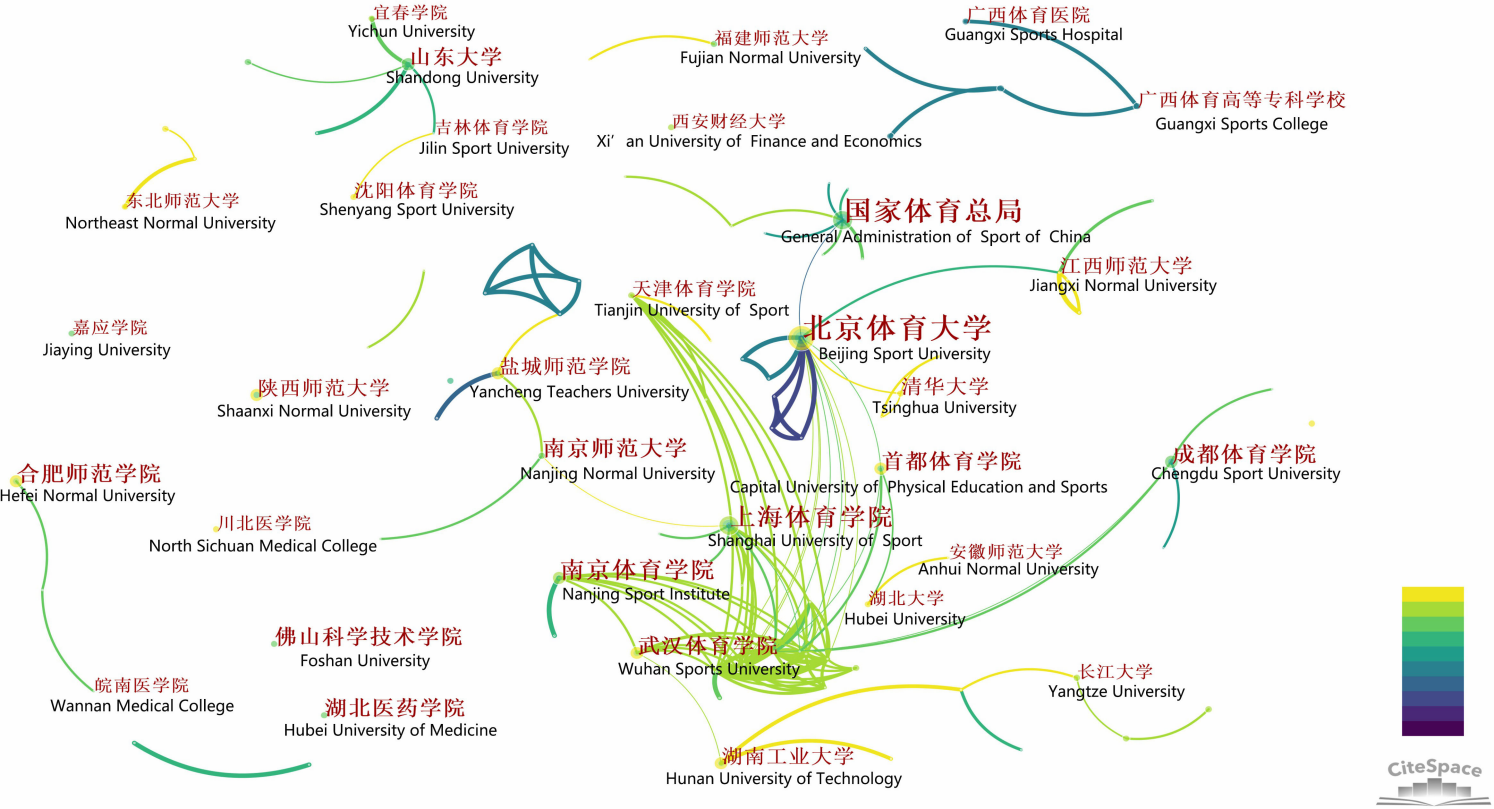
Bibliometric analysis of the institutions of publications on fusion of sports and medicine in China from 2012 to 2021. Each node represents a institution, the bigger the node the institution had more publications. The line indicated the relationship between institutions, the thicker the closer relationship, the lighter the later cooperation were formed. The English was translated from the original figure yielded by CiteSpace.

Furthermore, we tried to find out the leading institution of FSM research in China, and performed the analysis of institution distribution. The studies were conducted by 236 different institutions. Co-institution analysis showed that most scholars were from universities or colleges (*n* = 152, 64.41%), followed by hospitals or health center (*n* = 49, 20.76%) and other institutions such as high school, company and government departments (*n* = 35, 14.83%). The institutions had at least 10 publications in recent decade were Beijing Sport University (*n* = 20), Shanghai University of Sport (*n* = 13), Nanjing Sport Institute (*n* = 12), and General Administration of Sport of China (*n* = 10). The institutions had published more than five publications were shown in Figure 2. The bigger nodes indicated the more publications of institutions, and the line between them indicated a cooperative relationship. And we translated the original results (in Chinese) yielded by CiteSpace into English. The main cooperation network between institutions has formed, such as Beijing Sport University, General Administration of Sport of China and Capital University of Physical Education in North China, Wuhan Sports University and Hubei University in Central China, Nanjing Sport Institute and Nanjing Normal University in South China, Shanghai University of Sport in East China, and Chengdu Sport University in West China. Other smaller scale cooperation between institutions were within or in neighbor provinces, for example, Guangxi Sports College, Guangxi Sports Hospital and Guangxi University of Traditional Chinese Medicine (not shown) are all in Guangxi Zhuang Autonomous Region, Southwest China.

### Bibliometric analysis of the of journals and authors distribution

To find out which journal prefers research on FSM and has the most influence in China, we performed the bibliometric analysis of journal distribution. The 688 publications were published in 213 journals, and the journal with most publications on FSM was *Contemporary Sports Technology* (*n* = 74, 10.59%). The *China Sport Science* had the highest impact factor (IF = 6.468), which published 8 publications on FSM and had been cited 587 times till now. The top five journals with the highest frequency or impact factors of FSM were list in Table 2. The most-cited publication (130 times) was “Research on Connotation, Path, Institution and Mechanism of Deep Integration between National Fitness and National Health,” published in *China Sport Science* in 2018, and the authors were Lu Wen-Yun and Chen Pei-Jie from Shanghai University of Sport.

**Table 2:** The top five journals with the highest frequency and impact factors of fusion of sports and medicine.

**Rank**	**Journal**	**Frequency**	**Cited** **times**	**Impact** **factors**	**Journal**	**Frequency**	**Cited** **times**	**Impact** **factors**
1	Contemporary Sports Technology	70	143	0.202	China Sport Science	8	587	6.468
2	Science & Technology of Stationery & Sporting Goods	40	39	/	Sports Culture Guide	30	340	3.098
3	Sports Culture Guide	30	340	3.098	Journal of Wuhan Institute of Physical Education	10	166	2.917
4	Bulletin of Sport Science & Technology	26	67	0.395	Journal of Beijing Sport University	8	138	2.896
5	Sport Science and Technology	21	81	0.361	Journal of Chengdu Sport University	5	141	2.458

**Figure 3:**
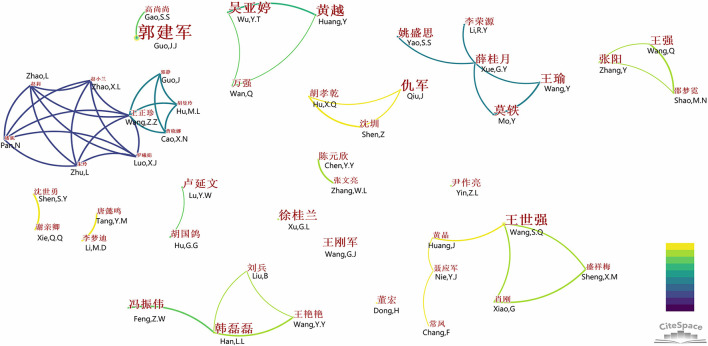
Bibliometric analysis of the authors of publications on fusion of sports and medicine in China from 2012 to 2021. Each node represents an author, the bigger the node the author had more publications. The line indicated the relationship between authors, the thicker the closer relationship, the lighter the later relationship were formed. The English was translated from the original figure yielded by CiteSpace.

To identify the author with the strongest influence and his/her research scope, the author distribution was conducted. The most prolific (12 publications) and most-cited (224 times) author was Guo Jian-Jun from Capital University of Physical Education and Sports. He focused on the research on the methods and development paths of the combination of sports and medical care under the “Healthy China” background. Huang Yue and Wu Ya-Ting from Hubei University of Medicine were the most prolific co-authors (eight publications), and they focused not only on the FSM education in university but also on the practice of FSM in youth and adolescents. Mo Yi from Guangxi Sports Hospital was the most prolific author from hospitals or health center (seven publications), and he focused on the health management in community residents, especially for those with chronic disorders (Table 3). Co-author analysis showed the author who had at least five publications, and some of them formed a cooperative network, such as a Beijing Sport University research team with Wang Zheng-Zhen as the center and a Guangxi Sports College research team with Xue Gui-Yue as the center (Figure 3).

**Table 3:** The authors of fusion of sports and medicine with more than five publications between 2012 and 2022.

**Rank**	**Name**	**Publications**	**Been cited**	**Year of first**
				**publication**
1	Guo Jian-Jun	12	224	2016
2	Huang Yue	8	51	2018
3	Wu Ya-Ting	8	51	2018
4	Mo Yi	7	42	2016
5	Han Lei-Lei	6	190	2018
6	Qiu Jun	6	44	2020
7	Wang Shi-Qiang	6	57	2019
8	Xu Gui-Lan	6	9	2020

### Bibliometric analysis of co-occurring keywords

**Figure 4:**
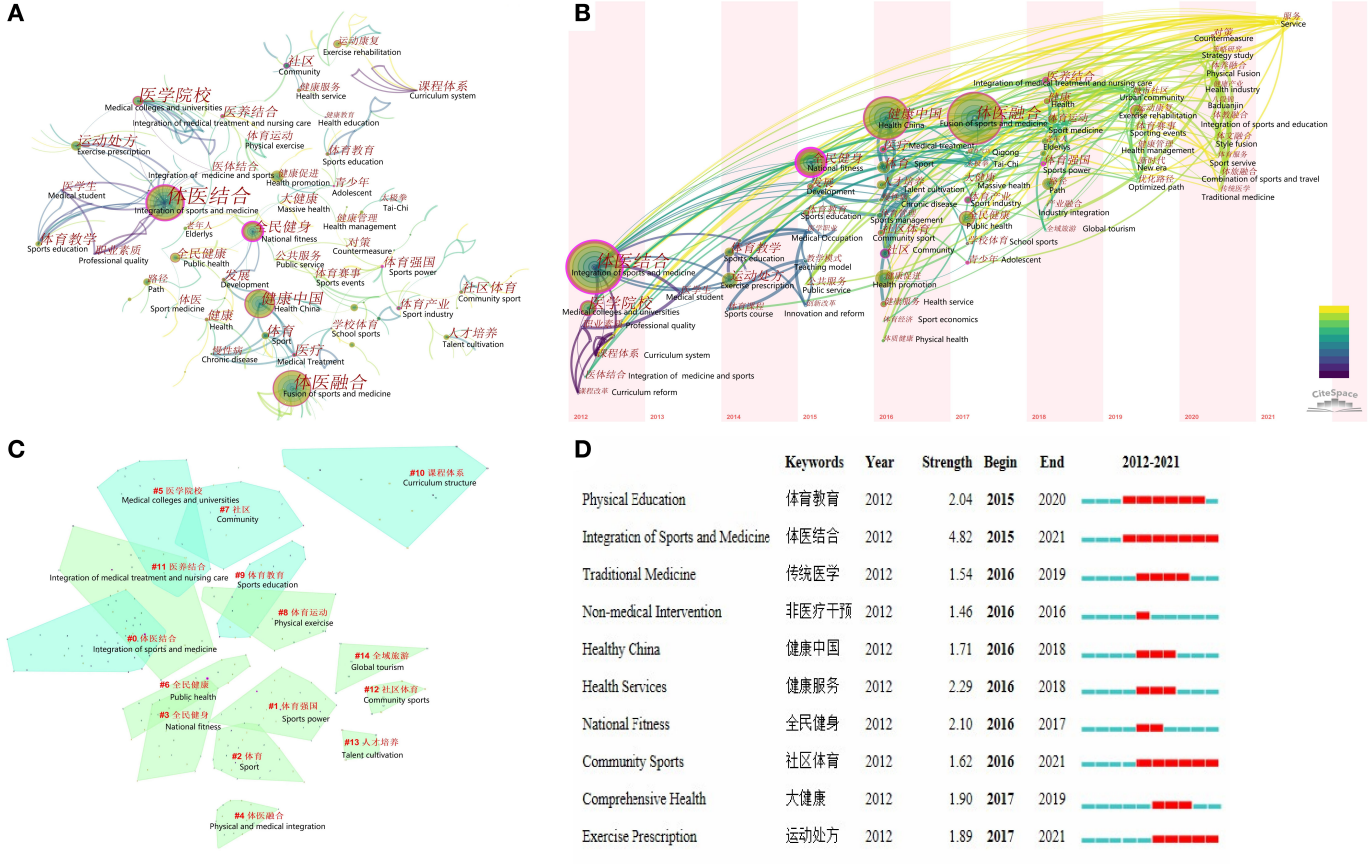
Bibliometric analysis of the keywords. **(A)** The network map of keywords from publications on fusion of sports and medicine in China; **(B)** A timeline view of the network map of keywords; **(C)** The 15 keyword clusters produced by log-likelihood ratio; **(D)** Top 10 keywords with the strongest citation bursts. Each node represents a keyword, the bigger the node the keyword had more appearance in publications, the node with purple trims indicated a high betweenness centrality. The line indicated the relationship between keywords, the thicker the closer relationship, the lighter the later relationship were formed. The English was translated from the original figure yielded by CiteSpace.

The occurrence of high frequency and high centrality of keywords show the focus of most authors in a period of time, that is, the hot spots and frontiers of research. The top three high frequency keywords were FSM, ISM, and Healthy China, while the top 3 high centrality keywords were national fitness, ISM, and sports power. Top 10 keywords with the highest frequency or the highest centrality were list in Table 4 respectively. As a high-level extraction of publication content, the keywords co-occurring can be calculated for specific fields in the context of publication, and the result, a network map of keywords, can reflect the past and current research hot spots and predict the hot spots in the future. The network map of keywords was showed in Figure 4A, each node represents a keyword in the publications, the node with purple trims indicated a high betweenness centrality. The line indicated a relationship between keywords, the thicker the closer relationship, and the darker the earlier the first co-occurring appeared. The lines around keyword ISM were darker than lines around keyword FSM, which indicated that the FSM showed up late and the relative research has just begun in recent years. A timeline view of the distinct keywords is shown in Figure 4B to present all the publications more clearly. The bold timeline indicates that the clustering topic was a hot spot during this period. The tree-rings with different sizes on the timeline represent some keywords with a high frequency. Then, we performed clustered network analysis to conduct a more in-depth study of those publications. There were 15 keyword clusters (all silhouette value >0.9) produced by log-likelihood ratio (Figure 4C). The silhouette value is used to evaluate the clusters, and the silhouette value above 0.7 is considering as the efficient and convincing cluster (7). The clusters were comprised with keywords with different colors, the overlap indicated the keyword belonged to two clusters at the same time. The Figure 4C showed that the cluster of #0 (ISM) contains the largest number of publications.

**Table 4:** The top 10 keywords of fusion of sports and medicine.

**Rank**	**Frequency**	**Keyword**	**Centrality**	**Keyword**
1	269	Fusion of sports and medicine	0.97	National fitness
2	191	Integration of sports and medicine	0.78	Integration of sports and medicine
3	126	Healthy China	0.43	Sports power
4	62	National fitness	0.42	Public service
5	32	Exercise prescrip tion	0.39	Healthcare
6	29	Medial universities or colleges	0.38	Healthy China
7	28	Health promotion	0.33	Fusion of sports and medicine
8	22	National health	0.32	Community
9	22	Sports	0.31	Sports industry
10	20	Pathway	0.30	Big health

Top 10 keywords with strongest citation bursts were presented in Figure 4D. The blue line indicates the time interval, while the red line indicates the time period when a keyword had a burst. These burst keywords were detected based on the increase in the frequency in the publications in that year, regardless of the total usage. Before 2015, the publications on FSM were rare, therefore, no keyword bursts before 2015. Since 2015, the research hot spots in FSM include physical education, physical medicine integration, traditional medicine, non-medical intervention, community sports, and exercise prescription, which indicated that the research on the training of talents for FSM was increased more than in previous years, and the scholars has gradually drawn attention to the research on teaching mode and curriculum system. Keyword “ISM” with the strongest bursts appeared in 2015 and continued to 2021, indicating that the Chinese scholars were inspired by the concept of “EIM” and became focused on this field. The most recent keywords with citation bursts occurred in 2017 and also continued to 2021 was “Exercise prescription,” the research mainly focuses on sports and clinicopathology, physical exercise and clinical manifestations, the therapeutic effect of physical exercise, the development and application of exercise prescription and other topics, which indicate that the concept of “ISM” had been translated into clinical practice in recent years.

## Discussion

### Summary of findings

The concept “FSM” in China was derived from the concept of EIM in the US. Between 2012 and 2021, there are 688 Chinese publications related to FSM screened out form CNKI database. In 2021, the number of publications reached the peek (*n* = 194). The authors of these publications were mainly from universities or colleges and most of them had sports background. The Beijing Sports University published the most publications on FSM (*n* = 20) and a cooperative network had taken shape in China. The most prolific (*n* =12) and most-cited (224 times) author was Guo JJ from Capital University of Physical Education Sports, however, the large cooperative network between authors had not formed yet. The journal with most publications on FSM was *Contemporary Sports Technology* (*n* = 74), while the *China Sport Science* had the highest impact factor (IF = 6.468) and 8 publications on FSM had been cited for 587 times. The analysis of keywords showed that the “FSM” had the highest frequency (*n* = 269), “ISM” had the strongest citation bursts (4.82), while “national fitness” had the highest centrality (0.97). Due to the short research time of FSM and insufficient cooperate between authors, 15 keyword clusters were produced and the studies frontiers seemed scattered.

### ISM, FSM, and health China

The top 3 most frequent keywords were ISM, FSM and Healthy China in recent decade. In chronological order, the concept of ISM was first reported in China in late twentieth century, after the issue of “*Healthy China* 2030” planning outline in 2016, the concept of FSM had became a hot topic in both academia and politics. However, the ISM and FSM were mixed used by scholars even till now. In 2019, the Chinese government issued the “'Build China into a Strong Sports Nation” outline, aimed to strengthen the national physical fitness and build China from a big sports nation into a strong one. Furthermore, in 2022, Ministry of Education announced that physical education had became one of the three major subjects for primary and middle school students. All these policies aimed to cultivate the habit of physical exercise from childhood among Chinese, reduce the medical burden and change from a treatment-centered health intervention model to a prevention-centered one. Therefore, the FSM seems to replace ISM under the “Healthy China” policy, and will be the highest guide to action in the future. However, to achieve the goal of FSM with Chinese characteristics, there is a long way to go, include the people, method and place.

### Universities and colleges, sports education, and talent cultivation

For a long time, the sports and medicine seemed to be irrelevant disciplines in China, except for sports medicine in medical schools (8). Both scholars with sports or medicine background had limit knowledge of each other. As the concept of FSM appeared, it is necessary to cultivate the talents with both backgrounds of sports and medicine. Since 2012, the research of ISM in universities and colleges had focused on curriculum reform, competitive sports, and the amount of exercise. The studies remained at the theoretical level and seldom put into practice especially for ordinary people. The keyword “physical education” had a citation burst between 2015 and 2020, which indicated that more educational institutions realized the importance of FSM and began to participate in the study of FSM. At that time, the research in sports universities and colleges focused on pathway research and curriculum optimization of physical education, and tried to cooperate with medical schools to cultivate interdisciplinary students. Moreover, some primary and middle schools began to participate in or conduct the research of physical education in children and adolescents. In order to cultivate interdisciplinary talents in a more standardized way, there are some suggestions. First, set up specialties related to FSM in universities or colleges, for example sports rehabilitation in sports schools and health management in medical schools. Second, establish a full system for FSM personnel to get training, evaluation, promotion, and continuing education. Third, provide courses in health management, basic medical treatment and first aid *etc*. for social sports instructors, meanwhile, provide courses in exercise prescription and sports intervention etc. for physicians or physical therapist. Fourth, learn from experienced countries or institutions, and introduce talents to guide the talent training programs in China.

### Traditional Chinese medicine, traditional Chinese exercise, and preventive treatment

The traditional Chinese medicine (TCM) and traditional Chinese exercise (e.g., tai-chi, baduanjin, yijinjing) are originated from the same theory. *Huangdi Neijing*, an ancient Chinese medical text, recorded that a good doctor performs preventive treatment rather than treating the already sick. Traditional Chinese exercise not only combined the active and static gestures together but also accompanied with breath training. In China, the elderly are more likely to accept TCM, and they are also the same population who susceptible to chronic diseases. Therefore, the participation of TCM helps to form FSM with Chinese characteristics. In 2016, “*Healthy China* 2030” planning outline recommended the use of traditional Chinese exercise for the prevention, treatment and rehabilitation of disease, especially for chronic diseases (3). Afterwards, scholars in China had paid more and more attention to the origin, concept, techniques, purpose, talent training and other aspects of traditional Chinese exercise (9, 10). Therefore, the fusion of sports (both modern sports and traditional Chinese exercise) and medicine (both modern medicine and TCM) will make more possibilities of health management for people in China.

### National health, national fitness, and community residents

The national health is a serious concern to the Chinese government, and national fitness is a relatively cheap and effective way to promote national health, while the community is the smallest unit capable of uniform health management (11). Our study found that the keywords “national health,” “national fitness,” and “community” were in the top 10 keyword of FSM list, and “national fitness” and “community sports” had strong bursts in last decade. Previous studies found that the community residents cannot acquire enough scientific sports guidance, had no enough sports equipment in community, the construction of network of national physical fitness surveillance was immature, and “sub-health” was difficult to get timely and effective surveillance. At present, there are three community FSM service models, featuring government-enterprise cooperation, community sports club, and community physical fitness surveillance center, respectively. Therefore, community health management is the combination of multiple strategies, including residential education, politics, policies, organizational support, living conditions and social conditions. In order to promote national fitness, the government should continue speeding up the promulgation of supporting policies and regulations, clarifying the responsibilities and relations between the administration departments, for example, integrating the superior resources of sports departments, health departments and schools at all levels, jointly formulating corresponding policies and systems, and deepening the national fitness plan into a national health plan.

## Strengths and limitations

To the best of our knowledge, this is the first bibliometric analysis on the China's FSM studies in English, and visual display by CiteSpace. The data downloaded from the CNKI database covered the vast majority of articles in the field of FSM research in China, and the data analysis was relatively objective and comprehensive, which clarified the past and present situation of FSM and predicted future research frontiers. However, this study also had some limitations. First, the concept of “ISM” and “FSM” was formed in 2016 and 2021, respectively, scholars have not reached consensus on the name of the concept in Chinese, let alone in English. Although there are hundreds of publications on the FSM studies in China in English database, we were unable to search them out by defined keywords. Therefore, the relevant publications might not be exhaustively identified. Second, because of the existence of multiple synonyms, there might be some overlap between different categories of content in the keyword clustering. Third, our study defined certain key words that may lead to data reduction. In addition, there was a time limit for our literature search. The first publication on “ISM” was published in 1998 in CNKI database, and the number of publication was small each year before 2012. Maybe it had limited effect on the findings of hot spots in recent research on FSM.

## Conclusion

Based on the CiteSpace findings, this study detected positive collaboration among institutions and authors. The core content of the FSM research and hot spots consistently related to the national policy guidance, industry development and social basic needs, indicating that theory and practice are still in progress of change. Main current research trends include the research on FSM for community residents, FSM talents training in schools and FSM pathway exploration with China characteristics. The study directions for scholars and policymakers in the future may include: improve the methodology system of the research on FSM; enrich the content of FSM; practice research on the mode of FSM; FSM mode with distinctive Chinese characteristics.

## Author contributions

S-JT, B-TC, and CL designed the study. S-JT, CJ, B-TC, and A-YX collected, analyzed, and visualized the data. S-JT and CL wrote the main manuscript text. CL and X-HW administrated the study. All authors contributed to the article, reviewed the manuscript and approved the submitted version.

## Funding

This work was supported by the National Natural Science Foundation of China (71974050 and 72274051).

## Conflict of interest

The authors declare that the research was conducted in the absence of any commercial or financial relationships that could be construed as a potential conflict of interest.

## Publisher's note

All claims expressed in this article are solely those of the authors and do not necessarily represent those of their affiliated organizations, or those of the publisher, the editors and the reviewers. Any product that may be evaluated in this article, or claim that may be made by its manufacturer, is not guaranteed or endorsed by the publisher.
